# Utilization of Dual Expandable Cages in Lateral Lumbar Interbody Fusion Surgery

**DOI:** 10.7759/cureus.41455

**Published:** 2023-07-06

**Authors:** Emmanuel Omosor, Brandon M Edelbach, Hammad Amer, Namath S Hussain

**Affiliations:** 1 Neurological Surgery, Loma Linda University School of Medicine, Loma Linda, USA; 2 Neurological Surgery, Loma Linda University School of Medicine, Redlands, USA; 3 Neurosurgery, University of California Riverside, Redlands, USA; 4 Neurological Surgery, Loma Linda University Medical Center, Loma Linda, USA

**Keywords:** minimally invasive spine surgery, lateral lumbar interbody fusion, lumbar spondylolisthesis, lumbar degenerative scoliosis, dual expandable cages

## Abstract

The aim of this study is to present a case series of adult patients with lumbar degenerative scoliosis who underwent focused minimally invasive spine (MIS) surgery utilizing a new dual expandable cage technology. The study investigates the effectiveness of this approach in reducing the symptoms and progression of lumbar degenerative scoliosis (LDS). Adult patients with lumbar degenerative scoliosis were selected for focused MIS using the newly introduced expandable cage technology. Patient demographics, preoperative evaluations, surgical details, and postoperative outcomes were recorded. The primary outcome measures included the restoration of disc space height, an improvement in clinical outcomes, and a reduction in surgical complications. Analysis of the case series reveals promising outcomes following focused MIS with the utilization of the new expandable cage technology. The technique demonstrated successful restoration of intervertebral disc space heights and improved clinical outcomes in patients with lumbar degenerative scoliosis. Furthermore, a notable reduction in surgical complications was observed. The findings from this case series suggest that MIS with the implementation of the new expandable cage technology holds promise for patients with lumbar degenerative scoliosis. This approach appears to have the potential to effectively restore disc space heights, improve clinical outcomes, and minimize surgical complications. Here, we want to emphasize and add details to the improved clinical outcomes of this technology; however, further research and larger prospective studies are warranted to validate these preliminary results and establish the long-term benefits and safety profile of this innovative technique.

## Introduction

Lumbar degenerative scoliosis (LDS) is a common pathology of unclear etiology that afflicts the elderly worldwide and is a cause of joint-related back and leg pain that degrades the quality of life [1,2]. Adult-onset degenerative scoliosis is a three-dimensional deformity described as a Cobb angle greater than 10° in the coronal plane [3]. LDS can have varied pathoanatomical abnormalities, such as stenosis, spondylolisthesis, and scoliosis, contributing to significant pain and disability [3]. Traditional surgical approaches to this disease often involve a combination of multilevel spinal decompression and vertebral fusion. Modern technological advancements in minimally invasive surgery (MIS) in conjunction with personalized patient care and appropriate patient selection have shown evidence for improved outcomes in this patient population [4,5]. Common MIS approaches include lateral lumbar interbody fusion (LLIF), anterior lumbar interbody fusion (ALIF), transforaminal lumbar interbody fusion (TLIF), and posterior lumbar interbody fusion (PLIF). These MIS approaches are reported to be associated with lower complication rates and morbidity, limited soft tissue disturbance, decreased blood loss, improved cosmesis, shorter hospital stays, and earlier return to work [5-8]. The use of dual expandable cages with percutaneous pedicle screw (PPS) placement in MIS spine surgery has been recently reported in the literature to provide restoration of intervertebral disc space height and alignment through a narrow operative window [7] but robust clinical data is lacking. Furthermore, there is a concern for subsidence of the endplates due to the point loading of a narrow cage [7]. In this study, we present a case series of adult patients with LDS who underwent focused MIS with the utilization of a new expandable cage technology with maintained disc space heights, improved outcomes, and reduced complications.

## Case presentation

Methods

We present five cases of lumbar degenerative scoliosis. Selection criteria included patients ages 18 to 80 with significant central and foraminal canal narrowing requiring discectomy. Furthermore, these patients had a history of failing conservative management. Arthrodesis was attempted with the dual expandable interbody spacer (Dual-X LLIF, Amplify Surgical, Inc., Irvine, CA) and pedicle screw instrumentation. These cases represent the use of a novel surgical implant for patient care and consent from the institutional review board was not needed. In this report, we examine the clinical presentation, surgical procedure, and radiographic data of each case as well as the possible benefits and constraints of this device.

The patient is positioned supine on the OR table and general endotracheal anesthesia is induced. Subsequently, the patient is repositioned in the prone position. The lateral bolsters are broken to laterally flex the spine. C-arm is used to identify the surgical level. Bilateral paramedian incisions are made in the lumbar spine based on fluoroscopic localization. Dissection is carried down to the bone. Any previous spinal instrumentation is inspected and removed. We then create a lateral spinal skin incision. Dissection is carried through the trajectory until the iliopsoas muscle and the transverse process of the vertebral body are palpable. The smallest tubular retractor is advanced from the lateral incision to the surface of the iliopsoas muscle. Its position over the indicated disk space is verified with fluoroscopy. It is then advanced through the iliopsoas muscle with neuromonitoring. Once this retractor is successfully docked on the disk, additional larger tubular retractors are advanced in a similar manner. The lateral lumbar interbody fusion (LLIF) retractor is then placed and opened. Soft tissue is cleared from the lateral edge of the disc with bipolar electrocautery. Discectomy is performed and an autograft is harvested. Upon satisfactory discectomy, we place our Amplify DualX expandable interbody spacer at the indicated disc space packed with autograft and allograft. The placement is confirmed using intraoperative fluoroscopy.

After irrigation, fascia, and skin closure, we proceed to use the C-arm to localize and place our pedicle screws. An integrated screw and K-wire are placed down into the indicated lumbar pedicles, with confirmation x-rays. Testing with intraoperative screw stimulation is performed to confirm all impedance thresholds are above 40 mA. At this point, if appropriate, a laminectomy is performed, and the decorticated bone is wrapped in Surgicel and placed down the gutters bilaterally. We then place two rods into the screw heads bilaterally and lock them with locking caps. After satisfactory final x-rays, all the incisions are closed.

Case 1

**Figure 1:**
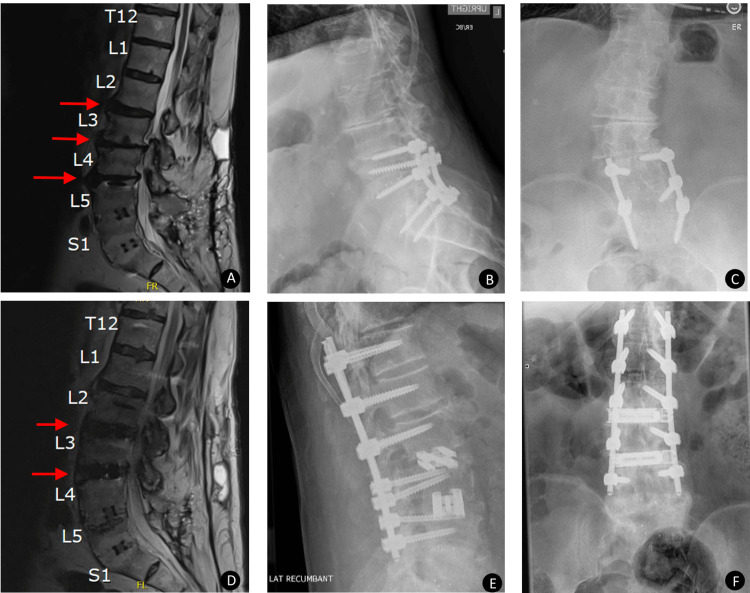
Case 1 postoperative radiographic evaluation of dual expandable spacer and screw placement A 73-year-old male presented with neurogenic claudication and low back pain. Preoperative sagittal magnetic resonance imaging (MRI) (A) demonstrating L3-L5 intervertebral disc desiccation and height loss (red arrows). Preoperative lateral (B) and anteroposterior (AP) (C) x-ray images showing left-sided predominant endplate osteophytosis, sclerosis, and disc height narrowing of L2-L4 with dextroscoliosis of the lower lumbar spine. Instrumentation from prior surgery is also visible. Postoperative sagittal MRI (D) showing postsurgical changes related to posterior spinal fixation hardware from T12-L4 with interbody spacers at L2-4 (red arrows). There is a minimally displaced L5 vertebral body fracture that abuts the previous hardware tracts. Postoperative lateral and AP x-ray images show intact spacers and maintained disc space height without subsidence (E, F).

A 73-year-old male, with a past medical history of myocardial infarction, congestive heart failure, stage two chronic kidney disease, and hypertension as well as a previous L4-S1 fusion, patient presented with complaints of lower back pain, lower left extremity weakness, and lumbar radiculopathy. His original injury was sustained in 1988 while lifting a heavy object. Imaging revealed stenosis of the lumbar foramina (Figures 1A-1C). He failed conservative management and had a progressive decline in functioning, which was affecting his quality of life. He was felt to be a good operative candidate and was consented for LLIF with pedicle screw fixation and fusion (Figures 1D-1F).

Case 2

**Figure 2:**
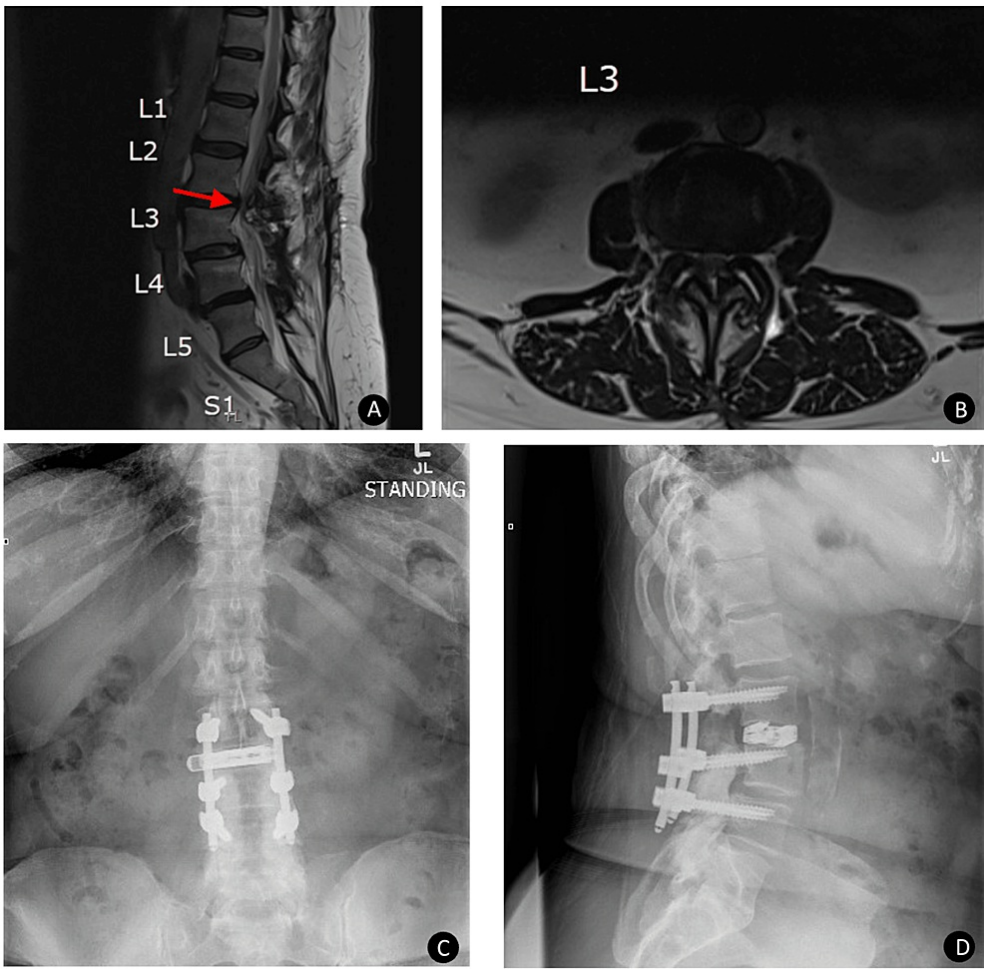
Case 2 postoperative radiographic evaluation of dual expandable spacer and screw placement A 70-year-old female presented with lumbar radiculopathy and low back pain. Preoperative sagittal (A) and axial MRI (B) showing postsurgical changes at L3-L4 from prior surgery with severe central canal stenosis at L2-L3 related to degenerative disc disease and facet arthropathy (indicated by the red arrow). Postoperative AP (C) and lateral (D) x-ray images show stable and intact posterior lumbar decompression and fixation from L2-L4 with intact interbody spacer and maintained disc space height.

A 70-year-old female with a past medical history of obesity and hypertension presented with low back pain and lumbar radiculopathy after a lifting accident. She had undergone a previous L3-4 fusion. Imaging revealed multilevel spondylosis most pronounced at L2-3 with foraminal stenosis (Figure 2A, 2B). She failed all conservative management and was felt to be a good operative candidate and was consented for LLIF with pedicle screw fixation and fusion (Figures 2C, 2D).

Case 3

**Figure 3:**
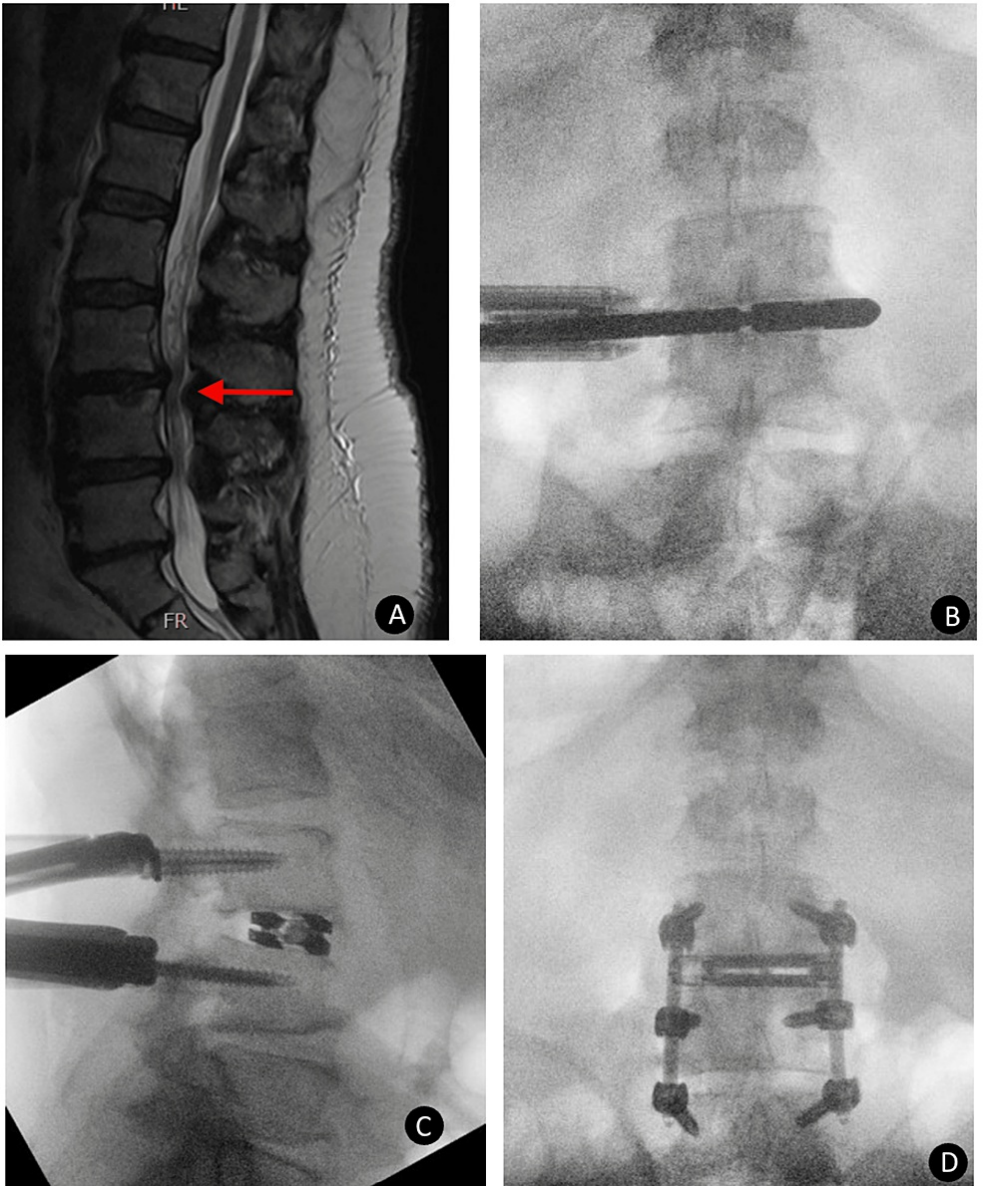
Case 3 postoperative radiographic evaluation of dual expandable spacer and screw placement A 69-year-old male presented with lumbar radiculopathy and low back pain. Preoperative sagittal MRI (A) showing multilevel degenerative changes of the lumbar spine with multilevel central spinal stenosis, severe at L3-4 (indicated by the red arrow). Intraoperative AP (B, C) x-ray image showing steps in the placement of posterior fusion hardware extending from L3-L5 with an interbody spacer at L3-L4. Postoperative AP (D) x-ray image showing posterior fusion hardware extending from L3-L5 with an interbody spacer at L3-4 with maintained disc space height and no subsidence.

A 69-year-old male, with a past medical history significant for obesity, diabetes, and hypertension, presented with a complaint of chronic lower back pain for multiple years but more recently numbness and weakness in his left lower extremity greater than the right lower extremity. The patient reported the symptoms occurred primarily when walking and worsened with prolonged distances as well as with sleeping. Imaging showed multilevel degenerative changes of the lumbar spine with multilevel central spinal stenosis, severe at L3-4, and multilevel neural foraminal narrowing (Figure 3A). He had tried conservative management without any durable benefit. He was felt to be a good operative candidate and was consented for LLIF with pedicle screw fixation and fusion (Figures 3B-3D).

Case 4

**Figure 4:**
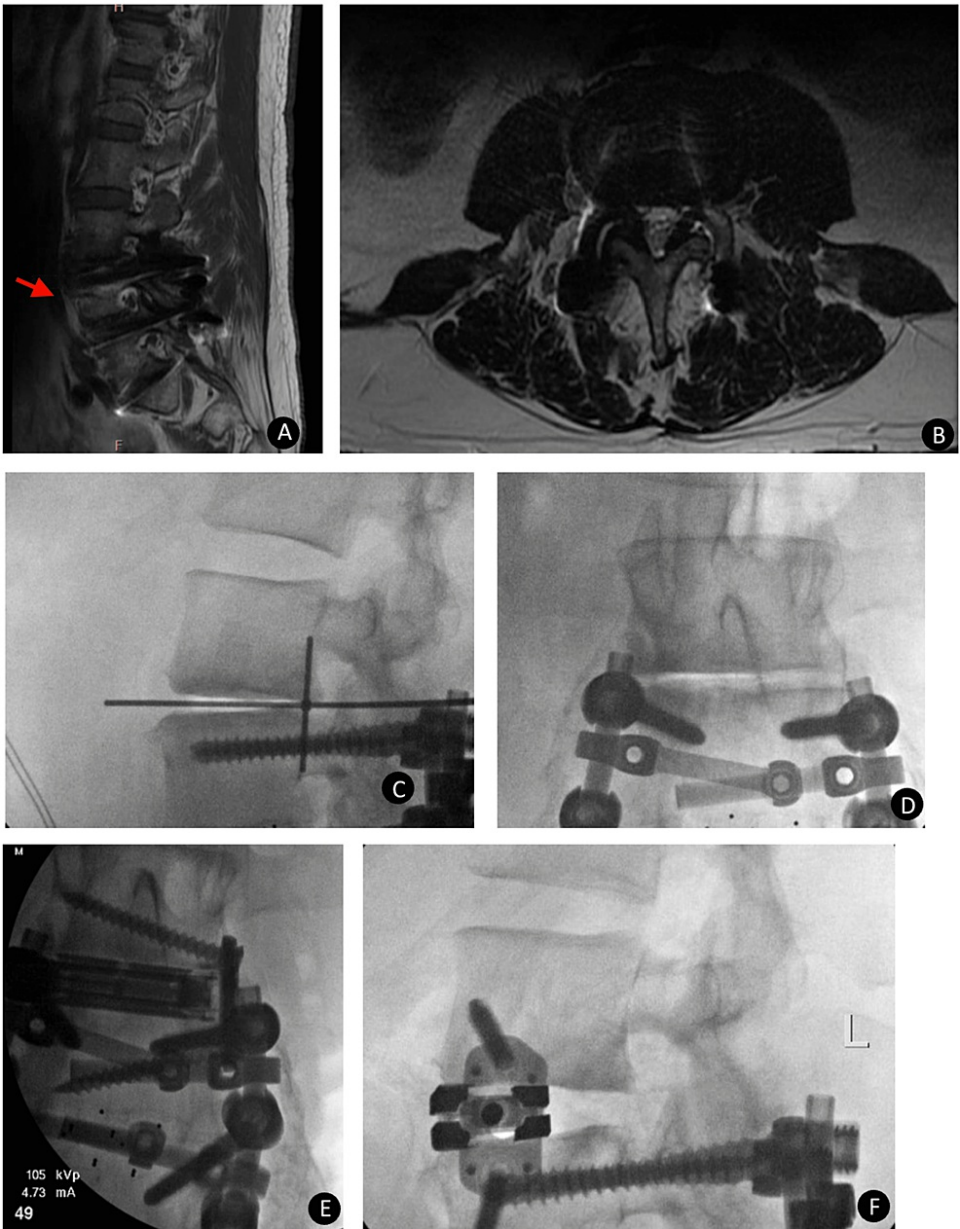
Case 4 postoperative radiographic evaluation of dual expandable spacer and screw placement A 55-year-old male with radiculopathy, neurogenic claudication, and low back pain. Preoperative sagittal (A) and axial (B) MRI showing L3-4 central canal and foraminal stenosis with severe adjacent level disc degeneration (red arrow). Intraoperative lateral (C) and anteroposterior (AP) (D) x-ray images showing steps in the placement of posterior fusion hardware. Intraoperative AP (E) and lateral (F) x-ray images show an expanded interbody spacer with maintained disc space height and no subsidence.

A 55-year-old male with no relevant past medical history presented with lower back pain and bilateral radiculopathy, both of which decreased his ability to stand upright and walk. His pain began in 1990 with no trauma history. He reports that he cannot walk very far and has weakness in his legs. The patient reported 50% of the pain is located in his back, and 50% of the pain is distributed through his legs. Imaging showed L3-4 spondylosis as well as severe foraminal stenosis (Figures 4A, 4B). He tried conservative management over the past several years with no durable benefit. He was felt to be a good operative candidate and was consented for LLIF with pedicle screw fixation and fusion (Figures 4C-4F).

Case 5

**Figure 5:**
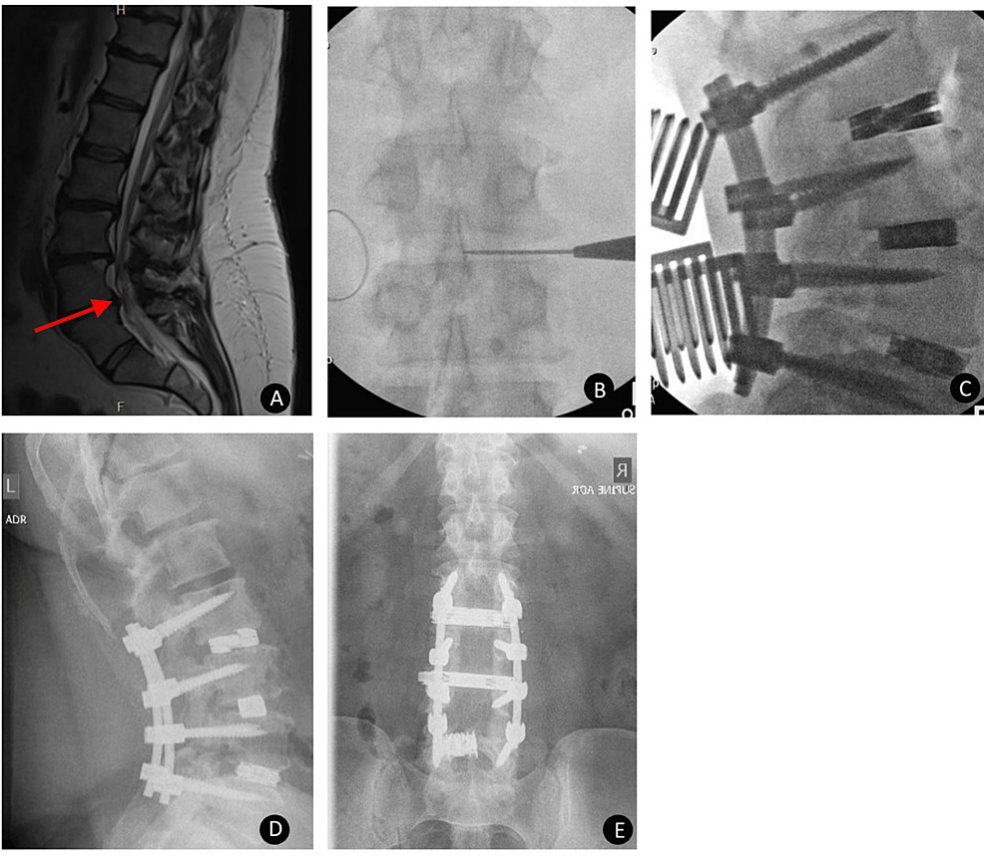
Case 5 postoperative radiographic evaluation of dual expandable spacer and screw placement A 53-year-old male with radiculopathy, neurogenic claudication, and low back pain. Preoperative sagittal MRI (A) showing moderate degenerative disc and facet disease at L3-S1 with moderate spinal stenosis (red arrow) at L4-S1 related to disk bulges, short pedicles, and prominent epidural fat. Intraoperative AP (B) and lateral (C) x-rays showing instrumentation and hardware placement along the lumbar spine. Postoperative lateral (D) and AP (E) x-ray images show intact interbody spacers without evidence of subsidence.

A 53-year-old female with a past medical history of chronic lower back pain presented with two days of acute onset numbness and weakness affecting the left lower extremity. MRI of the lumbar spine was concerning for moderate degenerative disc and facet disease at L3-S1, moderate spinal stenosis at L4-5 and L5-S1, as well as multilevel neuroforaminal narrowing, moderate at L4-5 and severe at L5-S1 on the left (Figure 5A). He was felt to be a good operative candidate and was consented for LLIF with pedicle screw fixation and fusion (Figures 5B-5E).

Results

We successfully performed LLIF surgery using a dual expandible cage in five patients (Table 1). All surgeries included bilateral decompression with LLIF and percutaneous pedicle screw fixation. The average age was 64 ± 8 years old with four males and one female. The diagnosis included lumbar spondylosis with central and foraminal stenosis (4 cases) and lumbar spondylolisthesis with central and foraminal stenosis (1 case). The levels involved included L2-L3 (3 cases), L3-L4 (5 cases), and L4-L5 (2 cases). Three cases required two-level fusions, and two cases required three-level fusions. The average operative time was 234 ± 144 minutes. The average estimated blood loss was 65 ± 43 mL. There were two complications: one patient had an epidural seroma causing right hip flexor weakness and numbness (G3 of 5) on postoperative day 19, post-discharge day 5 necessitating readmission for seroma evacuation with a consequent resolution of symptoms. Another patient had an L5 fracture adjacent to the fusion segment on postoperative day 14, post-discharge day 5 causing back pain and bilateral lower extremity weakness and pain. The patient improved after conservative pain management and was not reoperated. The intervertebral disc height space of the operative levels was significantly widened and maintained on postoperative radiographs without subsidence. The average disc height was significantly increased from 7.42 ± 2.0 mm to 13.92 ± 3.2 mm (p < 0.05) (Table 2).

**Table 1:** Characteristics of patients Values are presented as mean ± standard deviation.

Characteristics	Values
Age (year)	64 ± 8
Male to female ratio	4:1
Operation segment L2 – L3	3
Operation segment L3 – L4	5
Operation segment L4 – L5	2
Lumbar spondylosis with central and foraminal stenosis	4
Lumbar spondylolisthesis with central and foraminal stenosis	1
Mean operative time (min)	234 ± 144
Mean estimated blood loss (mL)	65 ± 43
Patients with Epidural seroma	1
L5 fracture adjacent to fusion segment	1

**Table 2:** Radiographic results Values are presented as mean ± standard deviation.

Variable	Preoperative	Postoperative
Disc height of operative segment (mm)	7.42 ± 2.033	13.92 ± 3.236

## Discussion

Historically, surgical intervention of LDS was focused on alleviating symptoms, with a large focus placed on neural decompression [9]. With the emergence of additional technology and MIS, treatment has evolved to include preventative measures such as stabilization and fusion. Of these correctional modalities, the interbody fusion technique was initially reported to have a high degree of success in fusions using bone grafting [10]. However, a significant degree of complications were later documented to occur with this procedure, including cauda equina syndrome and neurologic deficits [11-13]. A variety of surgical modalities (transforaminal, anterior, and lateral lumbar fusion) have since developed with the intent of lowering the associated symptoms of operation by providing stability.

One such advancement to address the stabilization and fusion of the vertebral body is the static cage. While this technology has advanced the treatment of LDS, this technology necessitates repeated trialing on the part of the surgeon and is associated with significant endplate disturbance from repeated insertion and extraction with is associated with deleterious effects [14]. Consequentially, the development of the dual expandable cage rose from the necessity of reducing the need to repeatedly trial and error with the static cage. This device shows promise in reducing patient post-operative symptoms through the utilization of the retroperitoneal, transpsoas approach to the disc space enabling MIS. This device is placed in the disc space in a collapsed state and then expands in both width and height which increases the surface area of endplate bony contact and allows contact with the apophyseal rings [7,15]. This permits customization of the height and width of the implant based on the patient's anatomy and specific needs. Adjustable height can help restore disc height and spinal alignment, which is essential for decompression and stabilization. Furthermore, the cage is designed to allow adjustment of the lordotic angle of the cage which is essential for maintaining a natural curvature, enhancing stability, and maximizing spinal alignment with the intent of decreasing post-operative symptoms and degenerative progression [7]. Lastly, the collapsibility of this device allows for a MIS approach, which when coupled with the lateral access technique of LLIF that is designed to reduce muscle trauma and spinal nerve disruption, resulting in significantly improved postoperative recovery.

The literature review of the use of expandable cages via the LLIF approach is limited. As highlighted in Table 3, this study corroborates with the existing literature illustrating significantly improved maintenance of disc space heights and patient-reported improvement in clinical symptoms. Of significance, Frisch et al. reported 27 such cases in 2018 and followed up for two years. Frisch did not report any significant difference in clinical outcomes when compared to static cages; however, radiographically, there was a significant difference in measured height with the static group maintaining a greater height at all follow-up points [16]. Li et al. reported 35 cases and followed up over two and found a significant improvement in leg pain when comparing an expandable spacer to a static spacer [17]. In 2020, Li et al. completed a similar study with a one-year follow-up on 37 dual expandable cage spaces via the LLIF approach. This study was limited in that it offered no control group; however, it did note an association with mild postoperative osteopenia [18]. In 2023, Huo et al. reported 48 patients receiving expandable cages with the expandable cage group notably having significantly higher rates of interbody fusion and reduced subsidence rates [19]. However, the impact of the dual expandable cage on subsidence rates is contested, as the titanium of the spacer may impact the degenerative progression of the vertebral body and contribute to subsidence, which is especially of concern in the context of osteopenic and osteoporotic bone [19,20]. Consequently, the use of expandable spacers is associated with increased fusion rates, and lower subsidence rates, both of which are independently associated with improved patient outcomes. However, the long-term adverse effects of this technology are not well-described and further data on clinical outcomes are necessary.

**Table 3:** Review and comparison of the relevant literature concerning MIS LLIF using expandable cages MIS: minimally invasive surgery; LLIF: lateral lumbar interbody fusion

Source	Number of patients (expandable spacer)	Number of patients (static spacer)	Mean patient age (expandable) (years)	Expandable operative time (min)	Static operative time (min)	Outcomes
Frisch et al. [16]	27	29	62.3±10.3	120.2±59.6	63.3±37.8	expandable subsidence lower than static group
Li et al. [17]	37	32	60.3±12.0	57.8±15.3	65.0±39.6	expandable subsidence rate lower than static and better clinical outcomes
Li et al. [18]	35	27	61.1±12.0	62.8 ± 24.3	66.9 ± 42.9	expandable subsidence rate lower and better clinical outcomes
Huo et al. [19]	48	50	71.2±8.5	Not reported	Not reported	expandable had higher fusion rates and lower subsidence rates
This study	5	0	64 ± 8.0	234 ± 144	Not reported	expandable subsidence rate lower with maintained disc space height and improved clinical outcomes

## Conclusions

In this study, a unique method was employed involving the use of a dual-direction expandable cage in minimally invasive lateral lumbar interbody fusion (MIS LLIF). To our understanding, this is the first documentation of such an approach in scientific literature. Preliminary observations from our research demonstrate encouraging radiographic and clinical results, as all implanted expandable cages exhibited effective preservation without significant collapse or subsidence. These initial findings suggest that the implementation of a dual-direction expandable cage in MIS LLIF may represent a feasible surgical option for the treatment of lumbar degenerative conditions.
